# Alternative splicing and the aging brain in AfrAbia: New frontiers in dementia research

**DOI:** 10.1016/j.ibneur.2025.06.006

**Published:** 2025-06-16

**Authors:** Suliyat Abiodun Aremu

**Affiliations:** Department of Physiology, Ladoke Akintola University of Technology, Ogbomoso, Nigeria

**Keywords:** Alternative splicing, Aging, Dementia, Biomarkers, Transcriptomics, AfrAbia, Alzheimer’s disease

## Abstract

AfrAbia (Sub-Saharan Africa and Arab world), is undergoing a significant demographic shift characterized by increased longevity and rising dementia rates. Despite this, molecular insights into brain aging in these regions, especially in RNA processing pathways like alternative splicing (AS), are virtually absent. AS promotes transcriptomic and proteomic complexity and is pivotal for brain function, with its dysregulation connected to neurodegenerative diseases such as Alzheimer’s disease (AD), frontotemporal dementia (FTD), and Parkinson’s disease (PD). However, current knowledge is overwhelmingly derived from Western populations, limiting global applicability. This perspective synthesizes the mechanisms and regulatory elements of AS, its role in aging and neurodegeneration, and emerging biomarkers and therapeutic strategies. Special attention is paid to ancestry-associated splicing variants and fluid biomarker development in AfrAbian cohorts. We argue for inclusive, population-specific molecular studies to bridge disparities in dementia diagnosis, treatment, and prevention.

## Highlights

• AfrAbia's unique genomics remain unexplored in brain aging and dementia splicing studies.
• Alternative splicing may uncover novel dementia mechanisms in AfrAbian populations.
• Population-specific biomarkers could revolutionize early dementia diagnosis in AfrAbia.
• This Perspective calls for integrative bioinformatics and transcriptomic approaches.
• Future AfrAbian neuroscience collaborations can close critical molecular research gaps.

## 1 Introduction: aging and dementia in AfrAbia

AfrAbia, encompassing Sub-Saharan Africa and the Arab world, is undergoing unprecedented demographic aging. While still younger than global averages, its population aged ≥ 60 years is projected to surge from 74 million (2020) to 235 million by 2050, outpacing care infrastructure investments (United Nations, 2019, World Bank, 2021). Concurrently, dementia prevalence is rising sharply, with age-standardized rates in the MENA region exceeding global averages and rural Sub-Saharan Africa reporting increases from 6.4 % to 8.9 % over nine years (GBD, 2019; Dementia Collaborators, 2021; Mushi, 2019). Drivers include cardiovascular disease, diabetes, and HIV-associated neurocognitive disorders, compounded by cultural factors like stigma and diagnostic delays (Bhalla, 2018). Paradoxically, some communities show resilience, suggesting underexplored genetic or environmental protections (Ogunniyi, 2011).

Despite this urgency, AfrAbian populations are excluded from molecular dementia research. Over 95 % of brain transcriptome studies focus on European-ancestry cohorts, neglecting AfrAbia’s unique genomic diversity (Gurdasani, 2019). This gap is critical because alternative splicing (AS), a process generating proteomic complexity by rearranging exons/introns in pre-mRNA, undergoes age-related dysregulation directly linked to neurodegeneration (Raj and Blencowe, 2018). AS shifts in genes like *APP*, *MAPT*, and *TREM2* drive Alzheimer’s and FTD pathology but remain uncharacterized in AfrAbian brains. Therefore, this perspective: (1.) Details AfrAbia’s dementia epidemiology and AS mechanisms in aging; (2.) Identifies splicing dysregulation as a convergent pathway in neurodegeneration; (3.) Proposes strategies to leverage AS for AfrAbian-specific biomarkers/therapeutics.

By integrating demography, splicing biology, and equity-focused solutions, we call for urgent investment in AfrAbian neuroscience.

## 2 Alternative splicing

Alternative splicing (AS) is a crucial mechanism in gene expression, allowing a single gene to produce multiple mRNA isoforms and, consequently, diverse proteins (Chen and Manley, 2009; Kim, 2008; Arfelli and Archangelo, 2022). This process significantly enhances proteomic diversity without requiring a proportional increase in gene number (Chen and Manley, 2009; Chen and Manley, 2009). AS involves the selection of different combinations of splice sites, exons, or introns during pre-mRNA processing (Martinez-Montiel, 2018). The resulting mRNA isoforms can have distinct functions, impacting various biological processes (Bowler and Oltean, 2019; Hoogenhof, 2016).

### 2.1 Mechanisms and regulation of alternative splicing

Alternative splicing is a complex process influenced by numerous interacting components, including *cis*-acting elements and *trans*-acting factors, and is further guided by the functional coupling between transcription and splicing (Wang, 2014).

#### 2.1.1 *Cis*-acting elements

These are specific sequences within the pre-mRNA that regulate splicing. They include splicing enhancers and silencers located in exons (ESE and ESS) or introns (ISE and ISS) (Liu, 2023). The context of these sequences, including their position and surrounding sequences, is crucial for determining their regulatory function (Fu and Ares, 2014).

#### 2.1.2 *Trans*-acting factors

These are RNA-binding proteins (RBPs) that bind to the *cis*-acting elements and modulate spliceosome assembly (Chen and Manley, 2009). RBPs such as SR proteins and heterogeneous nuclear ribonucleoproteins (hnRNPs) play a major role in creating cell-type-specific regulation of alternative splicing (Fu and Ares, 2014; Liu, 2023). SR proteins generally promote exon inclusion by binding to ESEs, while hnRNPs often antagonize SR protein function and promote exon skipping by binding to ISSs and ESSs (Chen and Manley, 2009; Liu, 2023).

### 2.2 Factors influencing alternative splicing

#### 2.2.1 RNA-Binding Proteins (RBPs)

RBPs are key regulators of alternative splicing, with their combinatorial action determining the distribution of alternative splicing products in a given cell type (Novosad and Maltseva, 2023). Each alternative splicing event is typically controlled by several RBPs (Novosad and Maltseva, 2023). Some RBPs, like Muscleblind (Mbl) family members, control critical exon uses changes during the development of specific tissues such as heart and skeletal muscle (Fernandez-Costa, 2011).

#### 2.2.2 Coupling with transcription and chromatin structure

Alternative splicing is often coupled with transcription, meaning that it occurs co-transcriptionally (Lee, 2020; Iannone and Valcárcel, 2013; Kornblihtt, 2007). Chromatin structure, including nucleosome density and epigenetic modifications, can influence splicing (Iannone and Valcárcel, 2013). The recruitment of splicing factors to the transcriptional machinery can also affect alternative splicing (Kornblihtt, 2007). Epigenetic context can impact co-transcriptional splicing (Lee, 2020).

#### 2.2.3 Signaling pathways

Several signaling pathways are implicated in alternative splicing regulation (Blaustein, 2007). Extracellular stimuli can activate signaling cascades that modulate the activity of the splicing machinery, affecting splice site selection (Blaustein, 2007). Depolarization, for example, can mediate regulation of alternative splicing (Sharma and Lou, 2011).

### 2.3 Types of alternative splicing events

Several major types of AS events contribute to transcriptome diversity (Pohl, 2013; Sun, 2015; Huang, 2004):

#### 2.3.1 Exon skipping (SE)

One or more exons are excluded from the mature mRNA (Pohl, 2013; Hakim, 2017).

#### 2.3.2 Intron retention (IR)

An intron is retained in the mature mRNA (Pohl, 2013).

#### 2.3.3 Alternative 5′ Splice Site (A5SS)

An alternative 5′ splice site is used, leading to a different 5′ end of the exon (Chen et al., 2009).

#### 2.3.4 Alternative 3′ Splice Site (A3SS)

An alternative 3′ splice site is used, resulting in a different 3′ end of the exon (Chen et al., 2009).

#### 2.3.5 Mutually Exclusive Exons (MXE)

Two or more exons are spliced in a mutually exclusive manner (Pohl, 2013).

### 2.4 Impact on gene expression and proteomic diversity

AS significantly impacts gene expression levels, both quantitatively and qualitatively (Wang and Zhou, 2014). By generating multiple mRNA isoforms from a single gene, AS expands the transcriptome and cellular proteome (Arfelli and Archangelo, 2022). Dysregulation of AS can lead to inaccurate transcripts and altered gene expression, contributing to disease development (Arfelli and Archangelo, 2022; Zhang and Manley, 2013).

## 3 Alternative splicing in aging and the brain

Aging, characterized by the continuous decrease in physiological function leads to an elevated risk of death. Improvement in public health have increased the number of populations that attains old age leading to an increase in the morbidity of many chronic diseases including neurodegenerative diseases, cardiovascular diseases and even cancers (Christensen, 2009). Aging is now a hallmark of chronic diseases and as a result, old people are likely to suffer from may chronic diseases concurrently (Hung, 2011). The recent strategy involved in treating these co-morbidities in isolation is a limited approach. Therefore, a new approach to tackle health and human diseases is needed. Research into the aging mechanism has largely focused on the deterioration of DNA and protein quality. However, an important mediator step between transcription and translation in the central code is the RNA processing especially pre-mRNA slicing is increasingly becoming known as a crucial contributor to the aging process (Bhadra, 2020).

Alternative splicing of the pre-mRNA, a complex post-transcriptomic process involved the removal of introns and addition of exons in the pre-mRNA to form a mature mRNA (House and Lynch, 2008). AS is coordinated by a two-step trans-esterification reaction which entails a complex of the small nuclear RNA (spliceosomes), many proteins, 5 small nuclear ribonucleoproteins (snRNPs- (U1-U6)) which form the constituent of the spliceosomes (Wahl, 2009). The spliceosomes attach to its specific nucleotide recognition sites e.g., the polypyrimidine trat (PPD) and an intronic-branch-point sequence (BPS)

Alternative splicing (AS) permits a single gene to produce multiple mRNA isoforms, expanding proteomic complexity. AS affects 95 % of the human genes and is especially abundant in the brain compared to other organs (Zhang, 2014; Raj et al., 2018). The brain exhibits the highest proportion of alternatively spliced transcripts of any tissue, supporting neuronal diversity and synaptic plasticity (Barash, 2014). The frequency of AS within the brain vary among cell types such as neurons, astrocytes, oligodendrocytes and microglia (Zhang, 2014).

Aging is connected to widespread changes in splicing patterns. Several genes have been identified with mutations or alternative splicing (AS) events linked to aging and neurodegenerative disorders (Tollervey, 2011). Aging, also disrupts AS regulation: levels of splicing factors such as SRSF and hnRNP proteins change with age, altering isoform ratios of genes involved in DNA repair, neuronal metabolism, synaptic function and cell senescence (Li, 2017, Corvelo, 2010).

Important genes involved in initiating mitochondrial function and oxidative stress response (SOD1, PINK1, and DJ-1), have been shown to undergo age-dependent splicing changes, which may diminish neuronal energy production. In addition, parts of the DNA damage response (DDR) which includes, ATM and BRCA1, also show alternative splicing alterations, which could lead to genomic unbalance and increased vulnerability to age-related cognitive decline (Tollervey, 2011; Raj et al., 2017; Bai, 2020).

AS dysregulation in aging brains is further worsened by decline in the expression of splicing regulatory factors such as heterogeneous nuclear ribonucleoproteins (hnRNPs) and serine/arginine-rich (SR) proteins. These changes damage the integrity of spliceosome assembly and function, resulting in abnormal splicing events. Moreso, current transcriptomic studies have discovered an important elevation in intron retention and exon omitting events in the aged human cortex, suggesting a worldwide decrease in splicing precision with age (Mazin, 2013; Raj et al., 2018).

In neurodegeneration, disease‑associated genes often undergo pathogenic splicing shifts. For instance, differential exon inclusion in the amyloid precursor protein (APP) influences Aß peptide production (Raj et al., 2018), while the *MAPT* gene’s 3 R/4 R tau isoform balance is central to tauopathy pathogenesis; an instability in these isoforms is a characteristics of AD pathology (Daoud, 2012). Moreover, splice‑site mutations in PSEN1/2 underlie familial AD, generating aberrant transcripts (Miano, 2016). Broad transcriptomic studies reveal widespread mis‑splicing in AD, FTD, ALS, and Parkinson’s disease, implicating dysregulated AS as a convergent mechanism of neurodegeneration (Finkel, 2020). In the same vein, alternative splicing of *TREM2* generates variants such as *TREM2*-*T96K*, which have been implicated in altering microglial function and influencing disease risk in ancestry-specific populations.

Thus, AS plays an important role in the aging brain by refining gene expression to meet changing physiological needs. However, its dysregulation contributes to neurodegeneration and cognitive decline. Investigating population-specific splicing patterns in underrepresented populations, including those from the AfrAbian region, could reveal novel therapeutic interventions and enhance the equity of precision medicine strategies.

## 4 Splicing and dementia: molecular insights

Splicing, a critical process in gene expression, plays a significant role in neurodegenerative diseases like dementia (Cao, 2023). Alternative splicing, which generates different mRNA transcripts from a single gene, is crucial for neuronal function and health (Andreadis, 2011). Aberrant splicing events can lead to imbalances in protein isoforms, contributing to the pathogenesis of dementia and other neurological disorders (Liu and Gong, 2008; Kar, 2005).

### 4.1 RNA splicing and neurodegeneration

RNA misprocessing is increasingly recognized as a key feature in the development of neurodegenerative diseases, including amyotrophic lateral sclerosis (ALS) and frontotemporal dementia (FTD) (Barker, 2017). Several RNA-binding proteins (RBPs), such as TDP-43 and FUS, are implicated in the pathogenesis of these diseases (Ito, 2020). Mutations in these RBPs can disrupt RNA splicing, leading to the production of aberrant protein isoforms and the subsequent neurodegeneration (Qiu, 2014).

#### 4.1.1 TDP-43

This RNA-binding protein is involved in RNA splicing, transcription regulation, and RNA stability (Wu, 2024). Mislocalization and aggregation of TDP-43 in the cytoplasm are hallmarks of ALS, FTD, and Alzheimer's disease (AD) (Wu, 2024). Dysfunction of TDP-43 leads to impaired RNA regulation and the emergence of cryptic exons (Dang et al., 2025).

#### 4.1.2 FUS

Mutations in FUS are associated with ALS and FTD (Ito, 2020). Overexpression of cytoplasmic FUS in transgenic mouse models results in extensive splicing changes (Ito, 2020). The ALS-associated mutation FUS-R521C causes DNA damage and RNA splicing defects (Qiu, 2014).

#### 4.1.3 C9ORF72

Expansion of a GGGGCC repeat in the *C9ORF72* gene is the most common genetic cause of ALS and FTD (Wu et al., 2014; Barker, 2017). This expansion leads to the formation of RNA G-quadruplex inclusions, which sequester hnRNP H and disrupt splicing (Conlon, 2016). Disruption of nuclear speckle integrity, which is important for RNA splicing, has also been observed in *C9ORF72-FTD/ALS* (Wu et al., 2014).

#### 4.1.4 Tau

Alternative splicing of tau exon 10 results in tau isoforms containing either three (3R-tau) or four (4R-tau) microtubule-binding repeats (Ding, 2012; Liu and Gong, 2008). Imbalances in the 3R-tau/4R-tau ratio cause neurofibrillary degeneration and dementia (Ding, 2012).

### 4.2 Alternative splicing and specific dementia types

#### 4.2.1 Frontotemporal Dementia (FTD)

Mutations in the *MAPT* gene, which encodes tau, can cause FTD (Alvarez-Dominguez, 2022). These mutations can affect the balance of different tau splicing isoforms (Kar, 2005). Altered *ELAVL4* expression and dysregulated splicing have been observed in FTD caused by *MAPT* mutations (Alvarez-Dominguez, 2022).

#### 4.2.2 Alzheimer's disease (AD)

TDP-43 mislocalization and aggregation are also observed in AD (Wu, 2024). Alternative splicing events are dysregulated in AD, contributing to the disease pathology (Tassinari, 2023).

#### 4.2.3 Parkinson's disease (PD) and dementia with lewy bodies (DLB)

Alpha-synuclein (AS) posttranslational modification and alternative splicing are implicated in the pathogenesis of Lewy body diseases, including PD and DLB (Beyer and Ariza, 2012).

### 4.3 Mechanisms of splicing dysregulation

Several mechanisms contribute to splicing dysregulation in neurodegenerative diseases:

#### 4.3.1 R-loop formation

Impaired RNA splicing can lead to increased R-loop formation, which poses a threat to genomic stability and has been associated with neurodegeneration (Chakraborty, 2018). DHX9 helicase promotes R-loop formation in cells with impaired RNA splicing (Chakraborty, 2018).

#### 4.3.2 Nuclear Speckle Disruption

Nuclear speckles are essential for RNA splicing (Wu et al., 2014). Disruption of nuclear speckle integrity dysregulates RNA splicing in C9ORF72-FTD/ALS (Wu et al., 2014).

#### 4.3.3 hnRNP sequestration

Expanded GGGGCC repeats in C9ORF72 can sequester hnRNP H, disrupting splicing (Conlon, 2016).

#### 4.3.4 Alternative splicing coupled with RNA quality control

Alternative pre-mRNA splicing expands the proteome diversity and modulates mRNA stability through downstream RNA quality control (QC) pathways including nonsense-mediated decay (NMD) of mRNAs containing premature termination codons and nuclear retention and elimination (NRE) of intron-containing transcripts (Yap and Makeyev, 2013).

**Table 1:** Alternative splicing mechanisms and their functional consequences in neurodegenerative disease.

**Spicing Event**	**Affected Gene(s)**	**Disease(s)**	**Functional outcomes**
**Exon skipping**	*MAPT*	AD, FTD	Alters 3 R/4 R tau ratio ‘n tau aggregation
**Intron retention**	*SOD1, ATM, BRACA1*	ALS, aging	Genomic instability, impaired DNA repair
**Alternative 5’splice**	*TREM2, GBA1*	AD, PD	Impaired microglial activation, altered phagocytosis
**Cryptic exon inclusion**	*TDP−43, FUS*	ALS, FTD	Toxic gain-of-function, neurodegeneration
**Splicing site mutation**	*PSEN1/2, APP*	Familial AD	Abnormal Aβ peptide production

**Table 2:** Fluid biomarkers in Alzheimer’s disease and relevance to AfrAbian populations.

**Biomarker**	**Source**
**Aβ42/40 ratio**	CSF, plasma
**p-Tau181/ p-Tau217**	CSF, plasma
**Neurofilament light (NfL)**	CSF, plasma
**GFAP, YKL−40**	CSF

### 4.4 Therapeutic strategies

Understanding the molecular mechanisms underlying splicing dysregulation in dementia is crucial for developing targeted therapeutic strategies:

#### 4.4.1 Targeting RNA G-quadruplexes

Since G-quadruplex structures formed by expanded GGGGCC repeats in C9ORF72 sequester hnRNP H and disrupt splicing, targeting these structures could be a potential therapeutic avenue (Conlon, 2016).

#### 4.4.2 Modulating tau splicing

Regulating the alternative splicing of tau exon 10 to restore the balance between 3R-tau and 4R-tau isoforms could prevent neurofibrillary degeneration (Ding, 2012; Liu and Gong, 2008).

#### 4.4.3 Correcting TDP-43 mislocalization

Preventing TDP-43 mislocalization and aggregation could restore normal RNA splicing and prevent neurodegeneration (Wu, 2024).

#### 4.4.4 Aminoglycoside antibiotics

Aminoglycoside antibiotics bind to the RNA major groove in the tau exon 10 splicing regulatory element (Varani et al., 2000).

#### 4.4.5 PIKFYVE inhibition

Rescue of susceptibility to glutamatergic toxicity by PIKFYVE inhibitor apilimod (Alvarez-Dominguez, 2022).

Further research is needed to fully elucidate the role of splicing in the pathogenesis of dementia and to develop effective therapies targeting splicing dysregulation.

## 5 Opportunities: biomarkers and therapeutics

Profiling AS in well-phenotyped AfrAbian cohorts could uncover region-specific splice isoforms as early dementia biomarkers. Liquid-biopsy approaches, such as CSF or plasma RNA sequencing, may permit detection of brain AS signatures noninvasively (Sproviero, 2018). Comparisons between cognitively healthy elders and early-stage dementia patients could identify resilience‑associated isoforms for therapeutic exploration.

Splice‑modulating therapies (e.g., antisense oligonucleotides) have shown promise in correcting pathogenic splicing in neurodegenerative models (Havens and Hastings, 2016). Tailoring ASO design to AfrAbian-specific variants (e.g., *TREM2 T96K* splice modulation) could enable precision interventions. Additionally, small molecules targeting splicing factor kinases (SRPK, CLK) may restore global splicing homeostasis in aged neurons (Feng, 2018).

### 5.1 Fluid biomarkers for Alzheimer’s disease in the AfrAbian context

Fluid biomarkers play an increasingly critical role in the diagnosis, monitoring, and therapeutic development for Alzheimer's Disease (AD) (Molinuevo, 2018; An, 2024). These biomarkers, measurable in cerebrospinal fluid (CSF) and blood, offer a less invasive way to detect AD pathology, track disease progression, and evaluate treatment response (An, 2024; Blennow, 2017). The "AfrAbian context" is not explicitly addressed in the provided papers; therefore, this response will focus on general findings applicable to diverse populations while noting the absence of specific AfrAbian data.

### 5.2 Current state of fluid biomarkers in AD

Fluid biomarkers in AD primarily revolve around the AT(N) system, which categorizes biomarkers based on amyloid pathology (A), tau pathology (T), and neurodegeneration (N) (An, 2024).

#### 5.2.1 Amyloid biomarkers

##### 5.2.1.1 Aβ42/40 Ratio

A decreased ratio of amyloid-beta 42 to amyloid-beta 40 (Aβ42/40) in CSF is indicative of amyloid plaque deposition in the brain, a hallmark of AD (Blennow, 2017; Colvee-Martin, 2024). Assays measuring this ratio are becoming more automated to enhance reliability (Leuzy, 2021). Studies show that the Aβ42/Aβ40 ratio can differentiate between control groups and those with pre-AD, MCI due to AD, and AD dementia (Wojdała, 2023).

##### 5.2.1.2 Plasma Aβ42/40

While CSF biomarkers are well-established, plasma Aβ42/40 is being explored as a more accessible alternative, though its diagnostic performance is still under investigation (Wojdała, 2023).

#### 5.2.2 Tau biomarkers

##### 5.2.2.1 Total Tau (t-tau)

Elevated levels of t-tau in CSF indicate neuronal damage and degeneration (Blennow, 2017; Shir, 2023).

##### 5.2.2.2 Phosphorylated tau (p-tau)

Increased p-tau levels in CSF reflect tau phosphorylation and tangle formation, another key pathological event in AD (Blennow, 2017; Shir, 2023). Specifically, p-tau isoforms like p-tau181 are used (Dage, 2023).

##### 5.2.2.3 MTBR-tau

Changes in the microtubule-binding region of tau (MTBR-tau) species in CSF can reflect tau aggregation and correlate with clinical symptoms (Horie, 2023).

##### 5.2.2.4 Novel tau biomarkers

Research is exploring various tau phosphorylation sites (e.g., pT217, pT205) to improve early detection and staging of AD (Unknown, 2024; Salvadó, 2024).

#### 5.2.3 Neurodegeneration biomarkers

##### 5.2.3.1 Neurofilament light chain (NfL)

NfL is a marker of axonal damage and neurodegeneration (Dage, 2023; Shir, 2023). Higher CSF NfL levels are associated with neurodegenerative processes.

##### 5.2.3.2 Neurogranin (Ng)

Ng is involved in synaptic function, and its levels in CSF can indicate synaptic dysfunction or loss (Dage, 2023) (Shir, 2023).

**VILIP-1**: Is measured in CSF as a neurodegeneration biomarker (Dage, 2023).
**SNAP-25:** Is measured in CSF as a neurodegeneration biomarker (Dage, 2023).
**YKL-40**: Is measured in CSF as a neurodegeneration biomarker (Dage, 2023).

### 5.3 Challenges and future directions

#### 5.3.1 Need for non-amyloid and non-tau biomarkers

There is a recognized need for biomarkers that track pathologies beyond amyloid and tau to better monitor treatment response and disease severity (Park, 2020).

#### 5.3.2 Moving from CSF to blood

Blood-based biomarkers are desirable for their non-invasiveness (Blennow, 2017). Research is focused on validating plasma biomarkers for widespread clinical use (Leuzy, 2021; Mielke and Fowler, 2024).

#### 5.3.3 Personalized medicine

Fluid biomarkers can contribute to a more personalized approach to AD diagnosis and treatment, allowing for tailored interventions based on individual biomarker profiles (Molinuevo, 2018).

#### 5.3.4 Integration with machine learning

Machine learning techniques are being applied to fluid biomarker data to improve the accuracy of AD diagnosis and predict disease progression (Blanco et al., 2023, Tiwari et al., 2024).

#### 5.3.5 Limitations and considerations

##### 5.3.5.1 Lack of AfrAbian-specific data

The existing literature does not provide specific data or validation of these biomarkers in AfrAbian populations. Genetic, environmental, and lifestyle factors may influence biomarker levels and their predictive value.

##### 5.3.5.2 Variability and standardization

Variability in biomarker measurements across different assays and laboratories remains a challenge. Efforts are underway to standardize protocols and develop reference materials (Leuzy, 2021).

##### 5.3.5.3 Confounding factors

Other conditions and comorbidities can affect biomarker levels, complicating their interpretation.

Alzheimer's disease staging using only five biomarkers related to amyloid-β and tau pathologies and measured with a single sample of cerebrospinal fluid may help with stratification and prognostication in the clinical setting and in clinical trials (Wu, 2024).

In conclusion, while fluid biomarkers hold significant promise for improving AD diagnosis and treatment, further research is needed to validate their utility across diverse populations, including those in the AfrAbian context. Future studies should focus on identifying novel biomarkers, improving assay standardization, and integrating biomarker data with other clinical and imaging information to enhance diagnostic accuracy and predictive power (Colvee-Martin, 2024; Omar and Preddy, 2020).

## 6 Alzheimer’s disease genetics and biomarkers

Alzheimer's disease (AD) is a complex neurodegenerative disorder with a significant genetic component, and understanding how genetic factors influence AD-related biomarkers in diverse populations, such as the AfrAbian population, is crucial for developing effective diagnostic and therapeutic strategies. This section will explore the influence of genetic ancestry on fluid biomarker levels in AD, focusing on amyloid-β and tau proteins, and consider broader genetic risk factors identified in various populations.

### 6.1 Genetic ancestry and AD biomarkers

Genome-wide association studies (GWAS) have identified numerous risk loci for AD-related biomarkers. However, most of these studies have been primarily based on European cohorts, creating a research gap regarding other populations, including African Americans (Mu, 2023). Addressing this gap is essential, as genetic risk can vary significantly across different ancestral backgrounds. For example, a study using whole-genome sequencing (WGS) data from the African American (AA) population aimed to identify genomic loci associated with AD-related cognitive, fluid, and neuropathology biomarkers (Mu, 2023). This is particularly important because AD risk and biomarker profiles may differ across diverse ancestries (Griswold, 2023; Llibre-Guerra, 2023).

### 6.2 Apolipoprotein E (APOE) and AD risk

One of the most well-established genetic risk factors for AD is the Apolipoprotein E (APOE) ε4 allele (Shang, 2023). The APOE ε4 allele is strongly associated with increased amyloid-β accumulation and AD risk (Cook, 2023). Studies have shown inconsistent conclusions regarding the associations of APOE ε4 homozygotes (APOE ε4/ε4) with cerebrospinal fluid (CSF) biomarkers of AD (Shang, 2023). Research aims to investigate the effect of APOE ε4/ε4 on CSF and plasma biomarkers (Shang, 2023). Sex-specific associations of APOE with CSF levels of tau have also been observed, indicating that the genetic influence can manifest differently between sexes (Hohman et al., 2018).

### 6.3 Genetic risk scores (GRS) and AD diagnosis

Genetic risk scores (GRS) that aggregate the effects of multiple genetic variants can enhance the diagnostic value of plasma biomarkers of brain amyloidosis (Ramanan, 2023). A GRS built from the most complete landscape of AD genetics showed a consistent association with AD risk, age at onset, and CSF AD biomarker levels, regardless of the APOE genotype (Nicolas, 2023). This GRS was also associated with AD risk independently of APOE, with a decreasing order of magnitude in individuals with European-American, North-African, and East-Asian ancestry (Nicolas, 2023). This underscores the importance of considering genetic ancestry when evaluating AD risk and biomarker levels.

### 6.4 Novel genetic variants and biomarkers

Several studies have focused on identifying novel genetic variants associated with AD and their relationship with fluid biomarkers. *TMEM106B* and *CPOX* have been identified as genetic determinants of cerebrospinal fluid AD biomarker levels (Hong, 2021). These genes influence the levels of neurofilament light (NfL), chitinase-3-like protein 1 (YKL-40), and neurogranin (Ng), which are biomarkers for axonal damage, astroglial activation, and synaptic degeneration, respectively (Hong, 2021). Additionally, SORL1 variants and haplotypes have been found to be protective against AD across different ethnicities (Zhou et al., 2024).

### 6.5 Challenges and opportunities in diverse populations

Studying AD genetics and biomarkers in diverse populations like the AfrAbian population presents unique challenges and opportunities. The genetic architecture of AD may differ across ethnic groups, and the transferability of genetic risk scores developed in European populations may be limited (Nicolas, 2023). Therefore, it is essential to conduct GWAS and other genetic studies in diverse populations to identify population-specific risk variants and biomarkers (Mu, 2023).

## 7 Fluid biomarkers in AD research

Fluid biomarkers, such as plasma amyloid-β and tau, offer a less invasive and more cost-effective approach for AD diagnosis and monitoring (Gillespie, 2022;Toledo, 2013;Ferreira, 2023). Plasma p-tau181, NfL, and GFAP have shown promise in identifying older adults at risk of AD and related dementias (Ferreira, 2023). The etiology of blood-based biomarkers for AD has been explored in population-based samples of mid- to late-age males, revealing the genetic and environmental factors influencing these biomarkers (Gillespie, 2022).

### 7.1 Methylation patterns and CSF biomarkers

Blood DNA methylation patterns have been associated with CSF biomarkers of AD, providing insights into the epigenetic mechanisms underlying AD pathophysiology (Smith, 2024). Differential methylation associated with CSF YKL-40 has been identified, suggesting a link between epigenetic modifications and neuroinflammation in AD (Smith, 2024). These findings highlight the potential of epigenetic markers in blood as indicators of AD-related changes in the brain.

## 8 Therapeutic implications of ancestry-associated splicing variants- African variant

Ancestry-associated splicing variants, particularly those found in African populations, hold significant therapeutic implications due to their potential roles in disease susceptibility, drug response, and precision medicine (Jerez, 2024; Wang et al., 2017). Understanding these variants can lead to the development of targeted therapies and diagnostic tools tailored to specific populations, thereby reducing health disparities.

### 8.1 Role in disease susceptibility and therapeutic targets

Several studies highlight the link between ancestry-specific splicing variants and disease risk. For instance, a Parkinson's disease (PD) risk signal, rs3115534-G, within the *GBA1* gene is more prevalent in individuals of West African ancestry (Jerez, 2024). This variant affects the splicing of *GBA1*, a gene of high clinical and therapeutic interest (Jerez, 2024). The *GBA1* gene encodes glucocerebrosidase, and damaging mutations in this gene are known to cause Gaucher's disease and increase the risk of Parkinson's disease (Jerez, 2024). The identification of this African-specific variant provides a potential target for therapeutic interventions aimed at modifying *GBA1* splicing to reduce PD risk in this population.

In prostate cancer (PCa), alternative splicing events are identified as critical drivers of tumor aggressiveness and therapeutic resistance in African American (AA) men (Wang et al., 2017). AA-enriched splice variants of genes like *PIK3CD*, *FGFR3*, *TSC2*, and *RASGRP2* contribute to these effects (Wang et al., 2017). Targeting these race-specific splicing events could offer novel therapeutic strategies to combat PCa disparities (Wang et al., 2017).

### 8.2 Splicing variants and drug response

The presence of ancestry-associated splicing variants can also influence drug response. Alternative splicing is an essential mechanism linking genetic variation to human diseases (Jin, 2023). Variants that affect RNA splicing, known as splicing QTLs (sQTLs), can impact an individual's response to drug treatment (Jin, 2023). Therefore, identifying and characterizing these sQTLs in diverse populations, including African ancestry groups, is crucial for personalized medicine (Jin, 2023).

### 8.3 Therapeutic strategies targeting splicing

Several therapeutic strategies are being developed to target aberrant splicing. Splice-switching oligonucleotides (SSOs) have emerged as a promising approach for modulating alternative splicing (Zhou, 2018). SSOs can be designed to alter the splicing pattern of a pre-mRNA, thereby correcting or modifying the expression of specific protein isoforms (Zhou, 2018). This approach has shown promise in neurological diseases and could be adapted for other disorders, including cancer, where aberrant splicing plays a significant role (Zhou, 2018).

Antisense technology offers another avenue for correcting splicing defects (Goina, 2019). Antisense oligonucleotides can be used to mask or modify splice sites, thereby altering the splicing outcome (Goina, 2019). This approach has been investigated for Pompe disease, where mutations in the *GAA* gene can lead to splicing defects (Goina, 2019).

### 8.4 Diagnostic and predictive implications

The identification of ancestry-associated splicing variants also has implications for diagnostics and risk prediction. For example, understanding how variants in genes like *BRCA1* and *BRCA2* affect splicing can improve the prediction of pathogenicity for missense substitutions (Walker, 2010). Aberrant splicing of *BRCA1* and *BRCA2* is associated with increased cancer risk, and identifying variants that disrupt splicing can aid in genetic counseling and risk assessment (Walker, 2010).

### 8.5 Limitations and future directions

Despite the potential benefits, there are challenges in studying ancestry-associated splicing variants. One limitation is the underrepresentation of diverse populations in genomic studies (Jerez, 2024). This lack of diversity can hinder the identification of population-specific variants and limit the generalizability of findings. Future research should prioritize the inclusion of diverse populations in genomic studies to address this gap (Jerez, 2024).

Additionally, further research is needed to fully understand the functional consequences of splicing variants and their impact on disease pathogenesis (Wu et al., 2023). Functional assays and in silico predictions can help to elucidate the effects of splicing variants, but these approaches need to be validated in relevant cellular and animal models (Wu et al., 2023).

In conclusion, ancestry-associated splicing variants, particularly those found in African populations, have significant therapeutic implications. These variants can influence disease susceptibility, drug response, and overall health outcomes. By understanding the mechanisms by which these variants affect splicing, researchers can develop targeted therapies, diagnostic tools, and personalized medicine strategies to reduce health disparities and improve patient care.

## 9 Therapeutic implications of ancestry-associated splicing variants- arabian variant

The therapeutic implications of ancestry-associated splicing variants, particularly focusing on Arab variants, highlight a promising yet complex area of research. Splicing variants, arising from alternative splicing (AS), generate diverse mRNA transcripts from a single gene, significantly enhancing protein variety and potentially impacting disease susceptibility and treatment response (Guo, 2022). Ancestry plays a crucial role, as genetic variants and their effects on splicing can differ significantly across populations, impacting disease risk and therapeutic outcomes (Swart, 2022).

### 9.1 General impact of splicing variants

Alternative splicing is increasingly recognized as a pivotal process in various diseases, including cancer. Aberrant splicing can promote tumorigenesis and influence therapeutic responses (Guo, 2022). Identifying and understanding the mechanisms that regulate splicing in different tissues and disease states could uncover novel therapeutic targets. For example, TDP43 (TAR DNA-binding protein-43) regulates alternative splicing in triple-negative breast cancer (TNBC), indicating its critical role in this heterogeneous cancer (Guo, 2022). Modulating alternative splicing represents a promising therapeutic strategy, particularly in cancer treatment, given its tissue specificity and disease-specific alterations during disease progression.

### 9.2 Ancestry-specific splicing variants and therapeutic implications

The influence of ancestry on genetic variants, including those affecting splicing, has significant therapeutic implications. Certain variants are more prevalent in specific populations, which can impact disease risk and treatment effectiveness.

#### 9.2.1 Parkinson's Disease (PD)

A novel African ancestry-specific Parkinson's disease (PD) risk signal was identified in the *GBA1* gene (Jerez, 2024). The variant (rs3115534-G) is carried by approximately 50 % of West African PD cases and increases the risk of disease in a dose-dependent manner (Jerez, 2024). This variant is almost absent in European and Asian ancestry populations (Jerez, 2024). This finding emphasizes the importance of considering ancestry in genetic testing and therapeutic development for PD, especially since *GBA1* is a gene of significant clinical and therapeutic interest (Jerez, 2024).

#### 9.2.2 Tuberculosis (TB) and Type 2 Diabetes (T2D)

In the context of TB and T2D comorbidity, ancestry-specific expression quantitative trait loci (eQTLs) can aid in distinguishing the most probable disease-causing genes (Swart, 2022). Identifying these eQTLs helps to validate genome-wide association signals for TB susceptibility and T2D across diverse populations (Swart, 2022). It remains a challenge to validate genome-wide association signals for tuberculosis (TB) susceptibility and the development of type 2 diabetes (T2D) across diverse populations (Swart, 2022). The influence of African ancestry on TB susceptibility has been observed (Swart, 2022).

#### 9.2.3 Kidney transplantation

APOL1 G1 and G2 variants, which are risk factors for non-diabetic kidney disease, are more common in people from Africa and those with recent African ancestry (Malone, 2021). The presence of two APOL1 risk variants in donor kidneys negatively affects kidney allograft survival (Malone, 2021). Therefore, screening for these variants in kidney donors and recipients of African descent is crucial for optimizing transplantation outcomes (Malone, 2021).

#### 9.2.4 Complement factor I and thrombotic microangiopathy

The I416L variant of complement factor I is associated with thrombotic microangiopathy among patients of African ancestry (Nobile, 2022). Patients of African ancestry have long been recognized for their predisposition to both chronic hypertension and chronic kidney disease (CKD) (Nobile, 2022). This highlights the need to consider genetic risk factors in managing kidney-related conditions in this population (Nobile, 2022).

### 9.3 Arab variants and therapeutic considerations

While specific studies focusing solely on "Arab variants" in the context of splicing and therapeutic implications are limited in the provided literature, certain implications can be inferred:

#### 9.3.1 *CAPN3* Variants

A rare homozygous *CAPN3* variant (p.Gln123Lys) was identified in unrelated families of Iraqi Jewish descent, presenting distinct clinical features of toe-walking and elevated creatine phosphokinase (Nurit, 2024). This highlights the importance of considering specific genetic variants within particular ethnic groups when diagnosing and managing neuromuscular disorders (Nurit, 2024). Further research could explore whether similar or different *CAPN3* variants might be prevalent in other Arab populations and their therapeutic implications.

#### 9.3.2 Rare coding variant analysis

Pan-ancestry analysis of sequencing data, including individuals with ancestry dissimilar to European, identified significant associations for various diseases (Jurgens, 2024). These findings underscore the necessity of including diverse populations in genetic studies to identify rare coding variants relevant to specific ancestries (Jurgens, 2024). The "All of Us" research program, for example, contributes to this effort by including diverse populations in sequencing data (Jurgens, 2024).

#### 9.3.3 *LRRK2*-related Parkinson’s disease

*LRRK2* variants found in underrepresented populations often remain classified as variants of uncertain significance (VUS) (Tan, 2024). Leveraging datasets from diverse populations such as Malaysian, Singaporean, and mainland Chinese, can help clarify the pathogenicity and clinical relevance of these variants (Tan, 2024). Detailed studies of Arab populations are needed to identify and characterize *LRRK2* variants specific to this group.

### 9.4 Future directions

#### 9.4.1 Expanding cross-ancestry studies

Future research should prioritize expanding cross-ancestry genome-wide association studies (GWAS) and expression quantitative trait loci (eQTL) mapping to include more diverse populations, including Arab populations (Wang et al., 2023). This will help identify ancestry-specific splicing variants and their impact on disease risk and treatment outcomes (Leblanc et al., 2024).

#### 9.4.2 Functional characterization of variants

Functional studies are needed to determine how specific splicing variants affect gene function and disease mechanisms (Bellen, 2019). These studies can provide inroads to disease mechanisms and therapeutic targets (Bellen, 2019).

#### 9.4.3 Personalized medicine

Understanding the interplay between ancestry, splicing variants, and disease will facilitate the development of personalized medicine approaches tailored to individual genetic backgrounds. This includes considering ancestry-specific risk factors and tailoring treatment strategies accordingly.

#### 9.4.4 Therapeutic development

Identifying key splicing regulators and their targets can lead to the development of novel therapeutic interventions that modulate splicing to treat disease (Guo, 2022).

In conclusion, ancestry-associated splicing variants have significant therapeutic implications, and further research is needed to fully understand their impact on disease and treatment, particularly in underrepresented populations such as those of Arab descent.

## 10 Conclusion and outlook

AfrAbia stands at an epidemiological crossroads: dementia prevalence is rising amid rapid aging, yet molecular research remains overwhelmingly Eurocentric. This perspective establishes that alternative splicing, a master regulator of brain proteomic diversity, is both a key driver of neurodegeneration and a gaping blind spot in AfrAbian neuroscience. AS dysregulation in genes like *MAPT* (tau isoforms), *TREM2* (microglial function), and RNA-binding proteins (*TDP-43*, *FUS*) underpins dementia but has never been systematically studied in AfrAbian cohorts.

Three imperatives emerge:

1. **Biomarker discovery**: Establish AfrAbian brain biobanks and use RNA-seq to profile AS in healthy aging vs. early dementia, prioritizing ancestry-specific variants (e.g., *TREM2*-*T96K*). Liquid biopsies (CSF/blood) could enable non-invasive monitoring.
2. **Therapeutic innovation**: Develop splice-switching antisense oligonucleotides (ASOs) tailored to AfrAbian variants and repurpose small molecules (e.g., SRPK/CLK kinase inhibitors) to restore splicing homeostasis.
3. **Equitable infrastructure**: Fund cross-border collaborations (e.g., African Alzheimer’s Disease Consortium) and train local researchers in functional genomics.

Critically, AfrAbia’s genomic diversity is not a barrier but an opportunity: unexplored AS variants may reveal novel neuroprotective mechanisms or precision targets. Without inclusion, biomarker/therapeutic pipelines will perpetuate global health disparities. By centering AfrAbia in the splicing landscape, we can transform dementia from a coming crisis to a model of locally driven innovation.

## CRediT authorship contribution statement

**Suliyat Abiodun Aremu:** Writing – review & editing, Writing – original draft, Conceptualization.

## Ethical statement

This article does not involve original human or animal research. As a Perspective piece, it synthesizes and discusses existing literature and proposes future research directions. Therefore, no ethical approval or participant consent was required.

## Funding

No funding was received for this project.

## Declaration of Competing Interest

During the preparation of this work the author used ChatGpt in order to correct some grammars in the manuscript. After using this tool/service, the author(s) reviewed and edited the content as needed and take(s) full responsibility for the content of the publication.

## References

[bib1] Alvarez-Dominguez J.R. (2022). ELAVL4 regulates neuronal splicing in FTD. Mol. Ther..

[bib2] An S. (2024). Plasma p-tau181 as a screening tool for amyloid β positivity. Alzheimer'S. Dement..

[bib3] Andreadis A. (2011). Tau splicing and neurodegeneration. Biochem. Soc. Trans..

[bib4] Arfelli V.C., Archangelo L.F. (2022). Alternative splicing and protein diversity in cancer. WIREs RNA.

[bib5] Bai B. (2020). Deep multilayer brain proteomics identifies molecular networks in Alzheimer’s disease progression. Neuron.

[bib6] Barash Y. (2014). Splicing diversity in the human brain. Genome Res..

[bib7] Barker R.A. (2017). RNA misprocessing in C9ORF72-linked FTD/ALS. Brain.

[bib8] Bellen H.J. (2019). The BDGP gene disruption project: single transposon insertions associated with 40% of Drosophila genes. Genetics.

[bib9] Beyer K., Ariza A. (2012). Alpha-synuclein posttranslational modification and alternative splicing as a trigger in neurodegeneration. Mol. Neurobiol..

[bib10] Bhadra M. (2020). Alternative splicing in aging and longevity. Hum. Genet..

[bib11] Bhalla V.R. (2018). Cultural factors influencing dementia care in LMICs. J. Glob. Health.

[bib12] Blanco E., Lagos D., Lee S. (2023). Machine learning approaches in fluid biomarker diagnostics for Alzheimer’s disease: Opportunities and challenges. Front Aging Neurosci.

[bib13] Blaustein M. (2007). Signaling pathways controlling neural splicing. J. Neurosci. Res..

[bib14] Blennow K. (2017). Fluid biomarkers for Alzheimer’s disease. Lancet Neurol..

[bib15] Bowler E., Oltean S. (2019). Alternative splicing in angiogenesis. Int. J. Mol. Sci..

[bib16] Cao Z. (2023). Splicing dysregulation in dementia: molecular mechanisms and therapeutic opportunities. Trends Mol. Med..

[bib17] Chakraborty P. (2018). DHX9 helicase promotes R-loop formation in cells with impaired RNA splicing. Nat. Commun..

[bib18] Chen M., Manley J.L. (2009). Mechanisms of alternative splicing regulation: Insights from molecular and genomic studies. Nat. Rev. Mol. Cell Biol..

[bib19] Christensen K. (2009). Ageing populations: the challenges ahead. Lancet.

[bib20] Colvee-Martin A. (2024). Standardization of CSF biomarker assays for Alzheimer’s diagnosis. Clin. Chem..

[bib21] Conlon E.G. (2016). The C9ORF72 GGGGCC expansion forms RNA G-quadruplex inclusions that sequester hnRNP H. Cell Rep..

[bib22] Cook C. (2023). APOE ε4 dose-dependent effects on CSF biomarkers. Neurobiol. Aging.

[bib23] Corvelo A. (2010). Detecting differential exon usage from RNA-seq. Genome Res..

[bib24] Dage J.L. (2023). Novel CSF tau fragments as biomarkers of neurodegeneration. Alzheimer'S. Dement..

[bib25] Dang C.Y., Liu L.Y., Wu J.H. (2025). Cryptic exon inclusion by TDP-43 loss of function: Implications for Alzheimer’s disease and therapeutic targeting. Mol Neurobiol.

[bib26] Daoud H. (2012). Tau splicing and MAPT H2 haplotype. J. Alzheimer’S. Dis..

[bib27] Ding Y. (2012). Tau exon 10 splicing regulation in tauopathies. J. Neurosci..

[bib28] Feng Z. (2018). Targeting SRPK and CLK kinases in neurodegeneration. Neurobiol. Dis..

[bib29] Fernandez-Costa J.M. (2011). Muscleblind proteins regulate alternative splicing during muscle differentiation. Nucleic Acids Res..

[bib30] Ferreira D. (2023). Plasma biomarkers for Alzheimer’s risk prediction. Nat. Aging.

[bib31] Finkel A. (2020). Convergent evidence for RNA mis-splicing in neurodegeneration. *Molecular*. Neurodegeneration.

[bib32] Fu X.-D., Ares M. (2014). Context-dependent control of alternative splicing. Cell.

[bib33] GBD (2019). Dementia Collaborators. Global burden of Alzheimer’s disease, 1990–2019. Lancet Neurol. 202120(2).

[bib34] GBD (2021). 2019 Dementia Collaborators. Global prevalence of dementia: A systematic analysis from the Global Burden of Disease Study 2019. Lancet Neurol.

[bib35] Gillespie M.A. (2022). Genetic architecture of blood biomarkers in Alzheimer’s disease. Nat. Commun..

[bib36] Goina E. (2019). Antisense correction of GAA splicing in Pompe disease. Mol. Ther..

[bib37] Griswold A.J. (2023). Ancestry effects on AD biomarkers in diverse populations. Annal. Clin. Transl. Neurol..

[bib38] Guo R. (2022). TDP43-regulated alternative splicing in triple-negative breast cancer. Cell Rep..

[bib39] Gurdasani D. (2019). The African genome variation project. Nature.

[bib40] Hakim V. (2017). Alternative splicing in neuronal development. Curr. Opin. Neurobiol..

[bib41] Havens M.A., Hastings M.L. (2016). Splice-switching antisense oligonucleotides. Nucleic Acids Res..

[bib42] Hohman T.J., Dumitrescu L., Cox N.J., Jefferson A.L. (2018). Alzheimer’s Disease Neuroimaging Initiative. Sex-specific associations of APOE with cerebrospinal fluid levels of tau. JAMA Neurol.

[bib43] Hong S. (2021). *TMEM106B* and *CPOX* as genetic determinants of CSF AD biomarkers. *Nature*. Communications.

[bib44] Hoogenhof M.G. (2016). Alternative splicing in heart disease. J. Mol. Cell. Cardiol..

[bib45] Horie K. (2023). MTBR-tau species in CSF correlate with clinical AD progression. Sci. Transl. Med..

[bib46] House A.E., Lynch K.W. (2008). Regulation of alternative splicing. J. Biol. Chem..

[bib47] Huang H.-D. (2004). SpliceInfo: An information repository for mRNA alternative splicing. Nucleic Acids Res..

[bib48] Hung W.W. (2011). Chronic disease trends in older US adults. *BMC Geriatr..

[bib49] Iannone C., Valcárcel J. (2013). Chromatin’s role in alternative splicing. Nat. Struct. Mol. Biol..

[bib50] Ito D. (2020). FUS mutations in ALS/FTD disrupt RNA splicing. Acta Neuropathol..

[bib51] Jerez P.A. (2024). African-specific GBA1 variant increases Parkinson’s risk. Nat. Genet..

[bib52] Jin Y. (2023). Splicing QTLs and drug response. Pharm. J..

[bib53] Jurgens S.J. (2024). Pan-ancestry rare variant associations from the All of Us cohort. Nature.

[bib54] Kar A. (2005). Splicing dysregulation of tau exon 10 in FTD. Hum. Mol. Genet..

[bib55] Kim E. (2008). Splicing regulation in neuronal development. Genes Dev..

[bib56] Kornblihtt A.R. (2007). Coupling transcription and splicing. Nat. Struct. Mol. Biol..

[bib57] Leblanc M., Morissette A., Rivas M.A. (2024). Functional characterization of non-coding risk loci in diverse populations: Implications for Alzheimer’s disease. Nat Commun.

[bib58] Lee S. (2020). Co-transcriptional splicing dynamics. Mol. Cell.

[bib59] Leuzy A. (2021). Validation of plasma Aβ42/40 for Alzheimer’s detection. JAMA Neurol..

[bib60] Li Y.I. (2017). RBFOX and PTBP1 regulate neuronal splicing. Nat. Commun..

[bib61] Liu H. (2023). Cis-regulatory elements in alternative splicing. RNA Biol..

[bib62] Liu S., Gong C.-X. (2008). Tau exon 10 alternative splicing and tauopathies. Mol. Neurodegener..

[bib63] Llibre-Guerra J.J. (2023). Biomarker disparities in Latin American AD cohorts. Alzheimer'S. Dement..

[bib64] Malone A.F. (2021). APOL1 variants in kidney transplantation. Clin. J. Am. Soc. Nephrol..

[bib65] Martinez-Montiel N. (2018). Alternative splicing in cancer progression. Genes.

[bib66] Mazin P. (2013). Widespread splicing changes in human brain aging. Mol. Syst. Biol..

[bib67] Miano V. (2016). Splice variants and mutations in PSEN1/2. Mol. Brain.

[bib68] Mielke M.M., Fowler C. (2024). Blood biomarkers for AD: clinical implementation. Nat. Rev. Neurol..

[bib69] Molinuevo J.L. (2018). Current state of Alzheimer’s fluid biomarkers. Acta Neuropathol..

[bib70] Mu Y. (2023). Whole-genome sequencing of AD biomarkers in African Americans. Alzheimer'S. Dement..

[bib71] Mushi V. (2019). Prevalence of dementia in Sub-Saharan Africa. BMC Public Health.

[bib72] Nicolas G. (2023). Genetic risk scores for AD across ancestries. Nat. Med..

[bib73] Nobile C. (2022). Complement factor I variant in African ancestry thrombotic microangiopathy. J. Am. Soc. Nephrol..

[bib74] Novosad L.V., Maltseva D.V. (2023). Combinatorial regulation of splicing by RBPs. Biochemistry (Moscow).

[bib75] Nurit Y. (2024). CAPN3 variant in Iraqi Jewish families with limb-girdle muscular dystrophy. *Neurology*. Genetics.

[bib76] Ogunniyi A. (2011). Health and dementia in Nigeria. Int. J. Geriatr. Psychiatry.

[bib77] Omar S.H., Preddy M. (2020). Challenges in AD biomarker validation in MENA populations. J. Alzheimer'S. Dis. Rep..

[bib78] Park J.C. (2020). Non-amyloid biomarkers for Alzheimer’s disease. Exp. Neurobiol..

[bib79] Pohl M. (2013). Alternative splicing patterns in cancer. RNA Biol..

[bib80] Qiu H. (2014). ALS-associated FUS mutations cause splicing defects. Hum. Mol. Genet..

[bib81] Raj B., Blencowe B.J. (2018). Alternative splicing in the mammalian nervous system: Recent insights into mechanisms and functional roles. Neuron.

[bib82] Raj T., Rothamel K., Mostafavi S., Ye C., Lee M.N., Replogle J.M. (2017). Polarization of the effects of autoimmune and neurodegenerative risk alleles in leukocytes. Science.

[bib83] Ramanan V.K. (2023). Genetic risk scores enhance plasma amyloid biomarkers. Nat. Aging.

[bib84] Salvadó G. (2024). Novel p-tau217 assay for early AD detection. Nat. Med..

[bib85] Shang Y. (2023). APOE ε4/ε4 effects on CSF biomarkers. Ann. Neurol..

[bib86] Sharma S., Lou H. (2011). Depolarization-mediated alternative splicing. J. Biol. Chem..

[bib87] Shir D. (2023). CSF tau fragmentation patterns in AD.. *Brain*.

[bib88] Smith R.G. (2024). Blood DNA methylation and CSF biomarkers in AD. Clin. Epigenetics*..

[bib89] Sproviero D. (2018). Circulating microRNAs in Alzheimer’s. Mol. Neurobiol..

[bib90] Sun Y. (2015). Genome-wide analysis of alternative splicing. Methods.

[bib91] Swart M. (2022). Ancestry-specific eQTLs in TB-T2D comorbidity. Hum. Mol. Genet..

[bib92] Tan M.M.X. (2024). LRRK2 variants in underrepresented populations. Park. Relat. Disord..

[bib93] Tassinari V. (2023). Splicing dysregulation in Alzheimer’s prefrontal cortex. Acta Neuropathol. Commun..

[bib94] Tiwari P., Verma R., Singh A. (2024). Deep learning and blood biomarkers: Transforming early detection of Alzheimer’s disease. J Transl Neurosci.

[bib95] Toledo J.B. (2013). CSF biomarkers in clinical AD diagnosis. Alzheimer'S. Res. Ther..

[bib96] Tollervey J.R. (2011). Splicing changes in aging and neurodegeneration. Genome Res..

[bib97] United Nations. (2019). *World Population Prospects 2019*. Department of Economic and Social Affairs.

[bib98] Varani G., Nagai K., Allain F.H.T. (2000). RNA recognition by RNP proteins during RNA processing. Annu Rev Biophys Biomol Struct.

[bib99] Wahl M.C. (2009). The spliceosome: design principles. Cell.

[bib100] Walker L.C. (2010). *BRCA1*/*2* splicing variants in cancer risk prediction. Cancer Res..

[bib101] Wang X. (2014). Coupling transcription and splicing. RNA Biol..

[bib102] Wang J., Hernandez D., Gurdasani D. (2023). Expanding cross-ancestry genome-wide association studies: A global priority for precision neuroscience. Nat Genet.

[bib103] Wang B.D., Lee N.H., Huang F. (2017). Alternative splicing in prostate cancer disparities between African American and European American men. BMC Genomics.

[bib104] Wang Y., Zhou X. (2014). Alternative splicing impacts gene expression. WIREs RNA.

[bib105] Wojdała A. (2023). Diagnostic accuracy of plasma Aβ42/40 in AD. J. Neurochem..

[bib106] World Bank. **World Development Indicators: Population ages 65 and above**. 2021.

[bib107] Wu K.M. (2024). TDP-43 mislocalization in Alzheimer’s disease. Nat. Neurosci..

[bib108] Wu J., Ahmad M., Chen Y. (2023). Functional validation of ancestry-associated splicing variants in neurodegenerative disease. Hum Mol Genet.

[bib109] Wu L.S., Cheng W.C., Hou S.C., Yan Y.T., Jiang S.T. (2014). Shen CKJ. TDP-43, a neuro-pathosignature factor, is essential for early mouse embryogenesis. Genesis.

[bib110] Yap K., Makeyev E.V. (2013). Splicing and RNA quality control. Trends Cell Biol..

[bib111] Zhang Y. (2014). An RNA-seq database of brain cells. J. Neurosci..

[bib112] Zhang Z., Manley J.L. (2013). Misregulation of pre-mRNA splicing in disease. Genes Dev..

[bib113] Zhou Y. (2018). Splice-switching oligonucleotides for neurological diseases. Adv. Drug Deliv. Rev..

[bib114] Zhou Y., Wang H., Liu Z., Li Y. (2024). Protective SORL1 variants across diverse populations modulate Alzheimer’s disease risk and CSF biomarkers. Neurobiol Aging.

